# Expression of the entire polyhydroxybutyrate operon of *Ralstonia eutropha* in plants

**DOI:** 10.1186/s13036-017-0062-7

**Published:** 2017-11-21

**Authors:** Rita Mozes-Koch, Edna Tanne, Alexandra Brodezki, Ran Yehuda, Ofer Gover, Haim D. Rabinowitch, Ilan Sela

**Affiliations:** The Robert H. Smith Faculty of Agriculture, Food and Environment, The Hebrew University of Jerusalem, 76100 Rehovot, Israel

**Keywords:** Polyhydroxybutyrate, “Green” plastic, Plant vector SE100, Chloroplasts

## Abstract

**Background:**

Previously we demonstrated that an entire bacterial operon (the PRN operon) is expressible in plants when driven by the Tomato -yellow-leaf-curl-virus (TYLCV) -derived universal vector IL-60.

Petroleum-derived plastics are not degradable, and are therefore harmful to the environment. Fermentation of bacteria carrying operons for polyhydroxyalkanoates (PHAs) produces degradable bioplastics which are environmentally friendly. However, bacterial production of bioplastics is not cost-effective, and attention is turning to their production in plants. Such “green” plastics would be less expensive and environmentally friendly. Hence, attempts are being made to substitute petroleum-derived plastics with “green” plastics. However, transformation of plants with genes of operons producing bioplastics has deleterious effects. Transformation of plastids does not cause deleterious effects, however it is a complicated procedures.

**Results:**

We have developed another TYLCV-based vector (SE100) and show that yet another bacterial operon (the phaCAB operon) when driven by SE100 is also expressed in plants. We employed the combination of SE100 and the phaCAB operon to drive the operon to the plastids and produce in plants a biodegradable plastic [polyhydroxybutyrate (PHB)].

Here we indicate that the bacterial operon (phaCAB), when driven by the newly developed universal plant vector SE100 is directed to chloroplasts and produces in plants PHB, a leading PHA. The PHB-producing plants circumvent the need for complicated technical procedures.

**Conclusion:**

The viral vector system SE100 facilitated the production of the bio-plastic poly-3-hydroxybutyrate. This was achieved by using the full pha-CAB operon indicating that TYLCV based system can transcribe and translate genes from bacterial operons controlled by a single cis element. Our data hints to the participation of the chloroplasts in these processes.

## Background

### Petroleum-derived plastics: general considerations

Plastics derived from petrochemicals are non-biodegradable (e.g., [26]) and considered environmental contaminants [11]. The potential of microbially- produced polyhydroxyalkanoates (PHAs) as biodegradable plastics has been investigated for decades. Biodegradable plastics can be produced in microorganisms, but cannot yet compete with petroleum-derived plastics mainly due to the latter’s more cost-effective production (e.g., [2, 14]). Driven by rising public awareness, the search for bioplastics with a “green agenda” is progressing [13], and the drive to merge current industrial practices with “green chemistry” is intensifying [20]. The diverse forms of PHAs (co-polymers and homopolymers) are suitable for a variety of possible applications: packaging materials, agriculture, medicine, cosmetics and more [1, 10]. A cost-effective production of degradable plastics would have great economic and environmental benefits.

### Expression of bacterial operons in plants

Prokaryotic genes are clustered in operons and their expression is driven by a common promoter. Operon-encoded proteins are translated from polycistronic mRNAs. Eukaryotic proteins are translated from their mRNAs, usually monocystronic, in a 5′-dependent manner [15]. Metabolic pathways in plants are determined by gene clusters rather than operons [5], requiring the coordinated expression of various genes. Artificially introducing these genes into plants to re-construct an entire metabolic pathway is quite difficult and requires selection and backcrossing of plants that express operon’s genes.

We previously reported the expression of an entire bacterial operon (the pyrrolnitrin [PRN] operon) in plants [17] under the control of IL60, a universal vector for expression/silencing in plants [19]. It is conceivable that operon expression in plants occurs in one of the prokaryotic-originated organelles (plastids/mitochondria). Preliminary studies indicated that PRN is produced in the chloroplasts. Here we demonstrate that another bacterial operon (the phaCAB operon) is expressed in plants under the control of a newly developed vector (SE100).

### The phaCAB operon

PHAs are produced by, and accumulated, in hundreds of species of bacteria, serving for intracellular carbon and energy storage. One of the major forms of PHA is poly-3-hydroxybutyrate (PHB). The biosynthesis of PHB in Ralstonia eutropha (formerly Alcaligenes eutropha) has been detailed. It is produced from acetyl coenzyme A (acetyl CoA) by a three-step reaction involving three enzymes (3-ketothiolase, acetoacetyl-CoA reductase and PHA synthase). The genes encoding these enzymes (PhaA, PhaB and PhaC) constitute the phaCAB operon [24]. Production of PHAs by fermentation of various bacteria and overcoming the various technical obstacles in the fermentation process has been attempted (e.g., [23, 25]). However, PHA production by fermentation requires costly investment in a carbon source and energy. This reduces its commercialization potential as the cost of production cannot compete with that involved in producing petroleum-derived plastics [16].

### PhaCAB expression in plants

Production of PHB in plants would be a more cost-effective strategy, as atmospheric CO2 serves as the carbon source and sunlight as the energy source. However, transferring components of the phaCAB operon, or the entire operon, into plants does not result in PHB production. Moreover, the phaCAB products, when present in the plant’s cytosol, hamper the plant’s survival and productivity. Expression of phaCAB in the plastome, however, is harmless [3, 4, 18, 22]. Transfer of phaCAB DNA to chloroplasts requires the construction of special vectors or the addition of chloroplast-directing signals to cytoplasm proteins.

The ability to express in plants entire operons, controlled by a single cis element, is of great value to plant biotechnology. It circumvents the need to transform plants separately with each separate gene (expressed at variable levels), then backcross the transgenic plants to reconstitute the entire metabolic pathway. Expression of operons has been reported in plants by directing the operon’s expression to the chloroplasts with special chloroplast vectors (e.g. [7] and references therein). Transformed chloroplasts are maternally inherited, minimizing (and in most cases abolishing) the transfer of foreign genes to the next generation or—by cross-pollination—to other crops. Thus, efforts have been directed toward plastid transformation [6]. The specific vector IL60, which directs expression in plants in a non-transgenic manner [19], can also drive the expression of an entire operon in plants [17]. Here we present data insinuating that the IL60-driven operon’s transcripts are directed to the chloroplasts.

## Results

### The TYLCV-IR may direct transcripts to chloroplasts

**Fig. 1:**
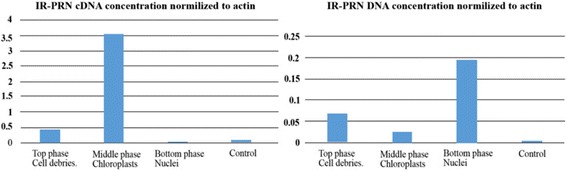
Detection of PRN cDNA and PRN DNA in subcellular fractions of host tomato plants transfected with IL-60-driven IR–PRN. Real-time PCR was carried out as described in Materials and Methods. Ordinates represent relative DNA or cDNA content. Total DNA (or cDNA) of untreated tomato plants served as a templates for the controls. PRN cDNA was reverse-transcribed from RNA of PRN-expressing plant

Reporter genes fused to the IR, driven by TYLCV-derived IL-60, can be expressed in plants [8]. The bacterial PRN operon driven by this system has also been expressed in plants [17]. We therefore postulated that the IR might participate in directing TYLCV to the (prokaryote-originated) chloroplasts. To that end we employed IL-60 to direct expression of the PRN operon, under the control of IR. IL-60 was co-introduced into tomato plants with the IR–PRN operon by the root-uptake method [8]. We postulated that the prokaryote-originated chloroplasts might be involved in operon expression. Therefore, subcellular fractions of the treated plants were separated by centrifugation on a Percoll density gradient. The presence of the DNA of the PRN operon and its transcripts (presented as cDNA) in the subcellular fractions of operon-expressing plants was determined by real-time PCR (Fig. 1).

**Fig. 2:**
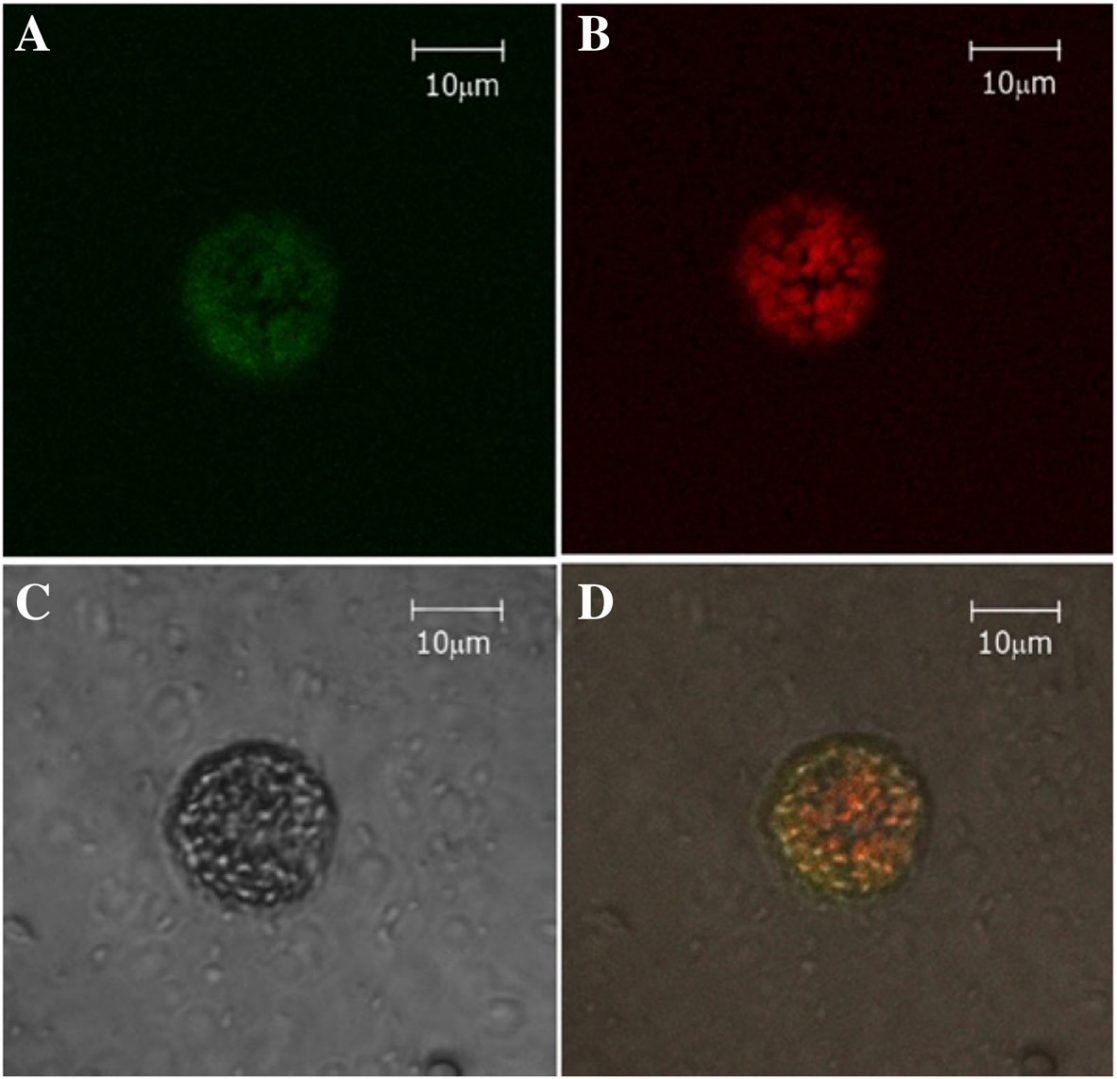
Confocal microscopy of protoplasts transfected with IR–PRN–GFP panels A, B, C and D depict (respectively): GFP fluorescence, chloroplast autofluorescence, no excitation, superimposition of panels A, B and C

Compatible with the consensus replicating cycle of TYLCV [9], the PRN DNA, driven by IL-60/IR, was detected mainly in the nuclei. However, cDNA of PRN was found in the chloroplasts (Fig. 1). The absence of IR-directed DNA and the presence of IR-directed transcripts in the chloroplasts excluded the possibility that (in the absence of a DNA template) the pertinent, non-chloroplast RNA was transcribed within the chloroplast. It is more likely that the IR (alone or with the aid of plant or viral factors) directed transcript mobilization to the chloroplasts. The indication that genes under the control of the IR are expressed in chloroplasts was corroborated by confocal microscopy. The gene encoding GFP was placed downstream of IR-PRN. Protoplasts were transfected with this IR–PRN–GFP. Confocal microscopy of the transfected protoplasts (Fig. 2) suggested that GFP is expressed in chlorophyll-carrying granular bodies (chloroplasts).

### Confirmation of successful uptake

**Fig. 3:**
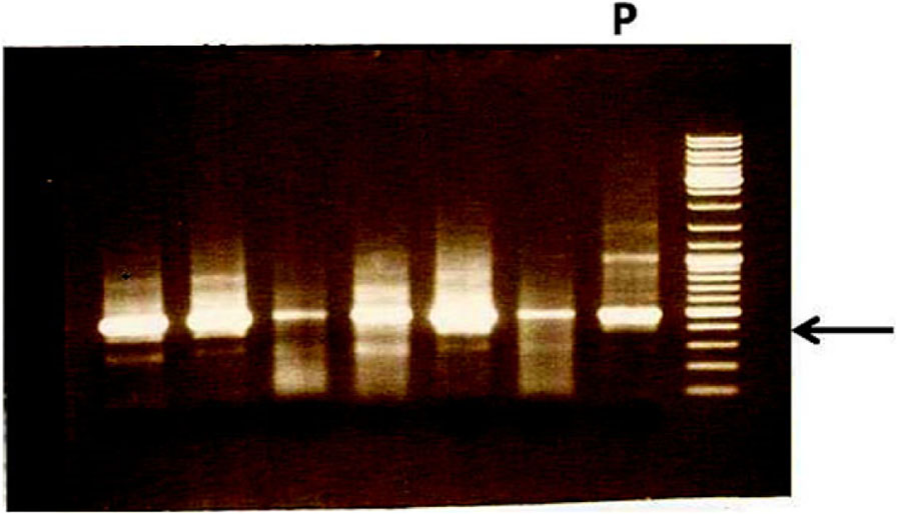
An electropherogram of PCR products amplified from phaCAB-treated plants. Each lane presents PCR results obtained from a different phaCAB-treated plant. P, positive control (amplification from a phaCAB-carrying plasmid). The arrow points to the position of the positive control

Following root uptake, the presence of *phaCAB* in plants was verified by PCR. DNA was extracted from leaves at various stages of development and served as the template for amplification. As shown in Fig. 3 *phaB* is present in the leaves of the growing plant, indicating the presence of the entire operon.

### The phenotype of phaCAB-carrying plants

**Fig. 4:**
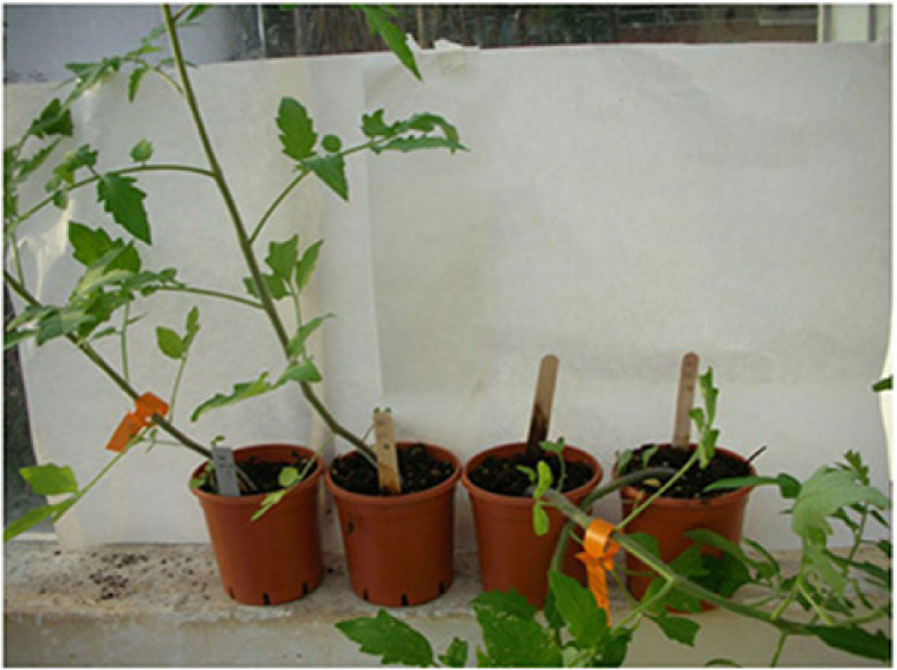
Untreated tomato plants (the two plants on the right) and phaCAB-expressing tomato plants (the two plants on the left), 1 month after planting of the treated seedlings

Under the employed conditions, untreated tomato plants grew upward and about a month after planting, the stems began to bend. Tomatoes expressing *phaCAB* remained erect at this stage and the stems bent only later, when they were longer. Figure 4 shows tomato plants at the stage at which untreated plant stems were bent over while *phaCAB*-expressing plants were still erect. Expression of *phaCAB* in the treated plants was confirmed by HPLC.

### HPLC verification of phaCAB expression

**Fig. 5:**
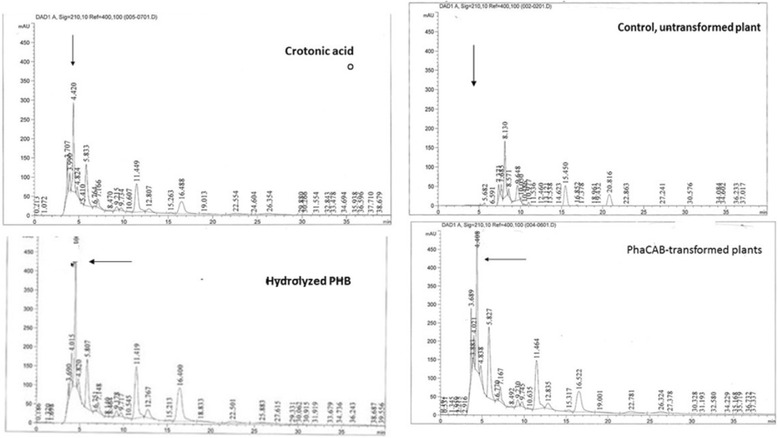
HPLC analysis of phaCAB-treated plants. A peak eluted at the retention time of crotonic acid (ca. 4.4 min) appears in hydrolysates of phaCAB-treated plants and not in control plants

Plants into which IR–phaCAB–pGOV4 was introduced along with SE100 were lyophilized and 50 g of the dry matter was hydrolyzed with concentrated H_2_SO_4_ [12]. The hydrolyzed plant material was diluted 1:1000 with 0.014 N H_2_SO_4_ (pH 2.7), and samples of 50–100 μL were submitted to HPLC analysis. Commercially available crotonic acid and PHB served as positive controls. The PHB was hydrolyzed prior to HPLC analysis. Figure 5 shows the presence of crotonic acid in the hydrolysates of *phaCAB*-treated plants but not control plants, indicating the presence of PHB. The HPLC analysis confirmed the expression of all of the *phaCAB* genes and production of the bioplastic PHB.

### GC–MS validation of the presence of crotonic acid in hydrolysates of treated plants

**Fig. 6:**
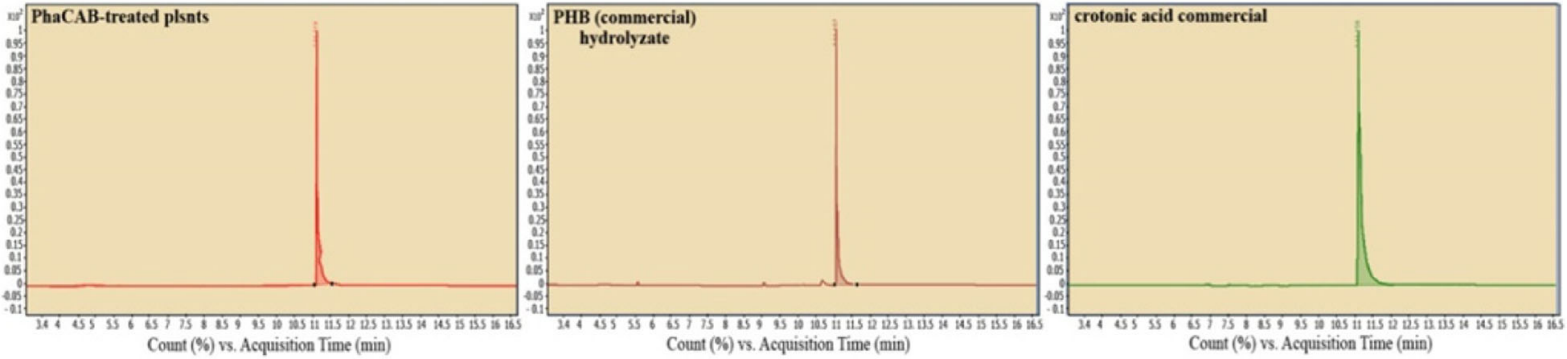
Presence of PHB in phaCAB-treated plants by GC–MS analysis

The presence of crotonic acid in the hydrolysates of *phaCAB*-treated plants was confirmed by GC–MS analysis (Fig. 6). The system was adjusted to detect compounds with a mass of 86 Da (the molecular mass of crotonic acid). The positive controls were commercial crotonic acid and PHB (hydrolyzed prior to GC–MS).

The MS results, in agreement with the HPLC results, clearly indicated that crotonic acid is present in phaCAB-treated plants.

## Discussion

We demonstrate that the entire phaCAB operon fused to the non-coding IR segment of TYLCV and driven by the SE100 vector is expressed in plants.

“Green” production of degradable plastics in plants has advantages over the production of the non-biodegradable petroleum-derived plastics, which are considered environmental contaminants. Many microorganisms produce bioplastics that are biodegradable [26]. However, production of microorganism-derived plastics is not cost-effective and cannot compete economically with petroleum-derived plastics.

Many research groups have turned their focus to the production of biodegradable plastics in plants. We have evidence that transcripts of the IR-PRN operon are located in chloroplasts, strengthening the notion that the (prokaryote-originated) chloroplasts are involved in operon processing in plants.

Compatible with the consensus replicating cycle of TYLCV [9], the PRN DNA, driven by IL-60/IR, was detected mainly in the nuclei. However, cDNA of PRN was found in the chloroplasts (Fig. 1). The absence of IR-directed DNA and the presence of IR-directed transcripts in the chloroplasts excluded the possibility that (in the absence of a DNA template) the pertinent, non-chloroplast RNA was transcribed within the chloroplast. It is more likely that the IR (alone or with the aid of plant or viral factors) directed transcript mobilization to the chloroplasts. The indication that genes under the control of the IR are expressed in chloroplasts was corroborated by confocal microscopy. Plants have been transformed with several individual genes that together constitute a pertinent bacterial operon, and “green” plastics have been produced by these plants. However, tedious procedures were required, involving the crossing of transgenic plants for several generations until all of the genes ended up in one plant. Since the expression of the inserted genes was not coordinated, further attempts were required to select a line of plants that produces the desired plastics. Moreover, transgenic plants that produce plastics in the cytoplasm are underdeveloped and suffer from impaired fertility (e.g. [3]). Thus, the expression of plastics in the plant cytoplasm is harmful [21], whereas their expression in plastids is less so, if at all [4]. In addition, transgenic plants are subjected to negative public opinion, and to regulations that minimize (or totally abolish) the possibility of using them. On the other hand, transformed chloroplasts are maternally inherited, minimizing (and in most cases abolishing) the transfer of foreign genes to the next generation or (by cross-pollination) to other crops. Thus, regulation of their broad use might not be as strict. Efforts have therefore been directed toward plastid transformation [6]. Chloroplast transformation requires the development of specific vectors [7]. The vector reported here (SE100) directs expression to plastids, circumventing many tedious procedures. Thus we present a straightforward procedure for the expression of entire operons in plants. In this study, we demonstrate the successful expression in plants of the bioplastic-producing operon phaCAB.

## Conclusions

The importance of this research is in showing the ability to express in plants entire operons, controlled by a single cis element. By using the TYLCV IR promotor to introduce foreign genes we show the production of bio-plastic in whole plants. This could be of great value to plant biotechnology.

## Methods

### Plants and constructs

Tomato plants (*Solanum lycopersicum*) were used throughout. The phaCAB operon of *Ralstonia eutropha* (GenBank accession no. AM260479, bases 1,557,353 to 1,561,203), modified to the codon usage of plants, was chemically synthesized (Gene Oracle, Mountain View, CA, USA), and placed in front of the Tomato yellow leaf curl virus intergenic region (IR; [19]). The entire synthetic construct was provided in a plasmid (pGOV4). The entire sequence of the IR–phaCAB–pGOV4 construct is described at the end of this section.

The construct of the SE100 vector was also chemically synthesized (Gene Oracle, Mountain View, CA, USA). The sequence of SE100 and the annotation of its various segments are also described at the end of this section.

Chloroplasts were isolated as follows: Leaves (10 g) were macerated in 150 ml GB buffer (20 mM HEPES pH 8.0, 300 mM sorbitol, 5 mM EDTA, 5 mM MgCl2, 5 mM EGTA, 0.05% BSA, 0.1% ascorbic acid), filtered through four layers of cheesecloth and centrifuged for 6 min at 6000 g. The pellet was gently suspended in 600 μl GB buffer. A Percoll step gradient (8 ml of 40% Percoll on top of a cushion of 4 ml 85% Percoll) was prepared in PBF (20 mM HEPES pH 8.0, 10 mM EDTA, 10 mM MgCl_2_, 1 g BSA, 1 g Ficoll, 3 g polyethylene glycol, 330 mM sorbitol) and the suspended pellet was loaded on top. Following 10 min centrifugation at 8000 g, the band in the middle of the gradient, consisting of chloroplasts, was collected and resuspended in SH buffer (50 mM HEPES pH 8.0, 330 mM sorbitol).

A nuclear marker sequence [REX-1; a *lycopersicum* marker for the root-knot nematode resistance gene Mi-l (GenBank accession no. AY589502.1)] was detected at the bottom of the gradient (the nuclear fraction) but not in the chloroplast fraction, whereas the chloroplast gene for 23S rDNA (GenBank accession No, KP119739.1) was detected in the chloroplast fraction but not in the nuclear fraction, demonstrating clear separation of chloroplasts from nuclei.

Chloroplasts were treated with RNase and DNase prior to extraction of their nucleic acids (Gröning et al., 1987; Schoelz & Zaitlin, 1989). They were then lysed by incubation (55oC, at least 4 h) in 8 ml lysis buffer (100 mM Tris-HCl pH 8.0, 100 mM NaCl, 50 mM EDTA, 1 mM DTT, 3.75% SDS, 30 μg Proteinase K). Following lysis, an equal volume of phenol: chloroform: isoamyl-alcohol (25:24:1) was added to the lysate and phases were separated by centrifugation. The nucleic acids in the aqueous phase were precipitated with isopropanol and the pellet was washed several times with ethanol.

The following primers were designed to amplify part of phaB: 5′-TAGTGAGGGCAATGTTGCAGAT-3′ and 5′-AGGACTTACTGTATTAACAGTTACCCCCTT-3′.

### HPLC analysis of PHB

The vector SE100 which replaces IL60 [19], drives the replication, expression and spread of any construct fused to IR. This vector was used to drive phaCAB expression in plants. The constructs were introduced into plants by root uptake: the root-tips of young tomato seedlings were clipped and placed in tubes containing SE100 and IR–phaCAB–pGOV4 (1 μg of each in H2O per plant) for 48 h until the entire solution was soaked up. The plantlets were then transferred to soil and allowed to grow. At several growth stages, the presence of phaCAB and SE100 was confirmed by PCR and ~2-month-old plants were taken for HPLC analysis of phaCAB.

PHBs are polymers of various sizes. Acid hydrolysis of PHB produces crotonic acid. Plant samples were lyophilized and acid-digested as described in Karr et al. [12].

Hydrolysates of samples of treated and control plants were diluted to 0.014 N H_2_SO_4_ (pH 2.7) and filtered through membranes. Samples of 5–50 μL were injected into an HPLC column (Agilent, Pursuit XRs C18, 250 × 4.6 mm). The mobile phase was 0.014 N H2SO4 (pH 2.7), and flow rate was 0.7 mL/min. Crotonic acid and PHB (Sigma Aldrich, Israel) served as positive controls. PHB was subjected to acid hydrolysis prior to HPLC separation.

### GC–MS analysis

Hydrolysates of phaCAB-treated plants and controls (crotonic acid and PHB hydrolysate, Sigma Aldrich) were analyzed by GC–MS. The gas chromatograph (Agilent 7890A) was coupled to a mass-selective detector (Agilent 5975C MSD) and equipped with a CTC COMBI PAL autosampler. Compounds were separated on a Stabilwax MS capillary column (30 m × 0.25 mm, 0.25 μm, Restek) using helium as the carrier gas at a flow rate of 1.3 mL/min. Sampling was accomplished using SPME (PDMS/DVB, Supelco) technique. The mass spectrometer was operated in positive EI mode. The system was adjusted to detect compounds with a mass of 86 Da (the molar mass of crotonic acid).

## Sequences of constructs

**The construct of the CAB-operon driven to plants by SE100.**

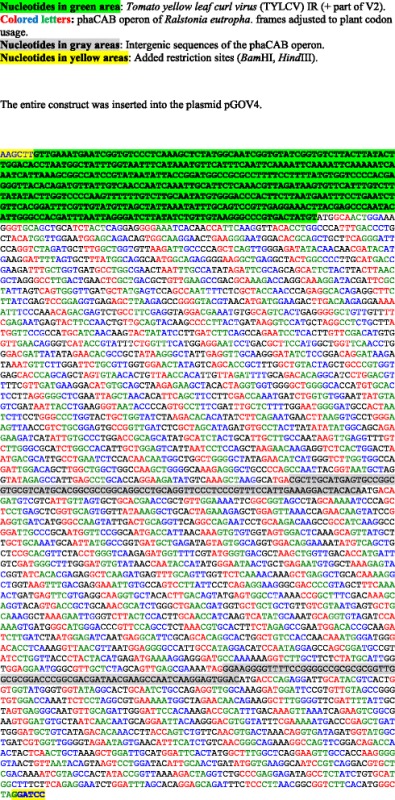



**The sequence and annotation of the vector SE100** (color coded).

SE100 was inserted into the plasmid pGOV4.
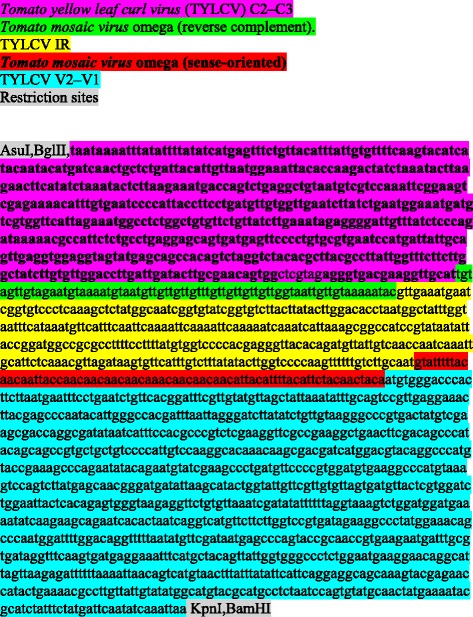


## Abbreviations

CP: Coat protein
IL-60: TYLCV-DNA after removal of 20 amino acids (positions 27–46) near the N terminus of TYLCV-CP
IR: Intergenic region
PHAs: Polyhydroxyalkanoates
PHB: Polyhydroxybutyrate
PRN: Pyrrolnitrin
TYLCV: Yellow-leaf-curl-virus
